# Effects of a Multicomponent Exercise Program on Groups of Community-Dwelling Older Adults with Low Schooling: A Pilot Study

**DOI:** 10.1155/2021/8829332

**Published:** 2021-06-03

**Authors:** Neildja Maria da Silva, Monalisa Silva de França, Dellis Kariny Freitas Holanda de Almeida, Evelin Suyany Guedes de Lima, Vinícius Hugley Brito dos Santos, João Victor de Araújo Souza, Ana Rodriguez Larrad, Débora de Almeida Aloise, Núbia Maria Freire Vieira Lima

**Affiliations:** ^1^Trairi School of Health Sciences (FACISA/UFRN), Santa Cruz, RN, Brazil; ^2^Department of Physiology, Bilbao, Basque Country, Spain

## Abstract

The aim of this study was to analyze the effects of a multicomponent exercise program on the physical and hemodynamic functions of community-dwelling older adults with low schooling levels in relation to simple multicomponent group exercises. Twenty-one older people were randomly assigned to two groups: G1 (*n* = 11) and G2 (*n* = 10); sixteen of whom completed the sixteen sessions over a six-week period, three times a week. During eight sessions, G1 performed adapted dual-task multicomponent exercises (strengthening, balance, and cognition) and G2 simple multicomponent exercises (strengthening and balance), and both groups engaged in eight additional sessions of simple multicomponent exercises. The dual-task multicomponent exercises exhibited similar effects to those of their simple multicomponent counterparts. The older adults from both groups improved mobility, frailty, static postural control, balance, and hemodynamic stability. The adapted program was beneficial to the community-dwelling older people with low schooling in the group intervention.

## 1. Introduction

According to the United Nations, the decline in fertility rates and increased life expectancy have resulted in an aging population worldwide. Projections made in 2015 estimate that the population aged 60 years or older will be 1.4 billion by 2030 and 2.1 billion by 2050 [1]. In Brazil, according to the sociodemographic data of the Brazilian Institute of Geography and Statistics, the population aged 60 years and older in 2000 was 14.2 million, rising to 19.6 million in 2010 and estimated to be 41.5 million by 2030 [2].

Aging triggers changes that compromise the physical and cognitive functions of older adults [3]. According to the American College of Sports Medicine guidelines [4], regular physical exercise increases life expectancy and decreases the harmful effects on age-related biological functions. Additionally, studies have demonstrated that exercise delays the emergence of chronic diseases and functional decline [5], prevents cognitive decline [6], delays the onset of neurodegenerative diseases [7, 8], and slows frailty progression [9]. A recent systematic Cochrane review of 23,407 older adults from 25 countries found that balance and gait training exercises and muscle strengthening lowered the rate of falls by up to 23% and the number of falls by 5% [10, 11].

Studies aimed at identifying the benefits of multicomponent exercise programs for older people observed positive effects on frailty and falling [12, 13], physical function [14, 15], cognition [16], and functionality [17], as well as a decline in blood pressure and heart rate [18]. In addition to multicomponent programs, other studies investigated the effects of dual tasks combined with multicomponent exercises in the older population and the results demonstrated improved mobility and balance [19–21], as well as physical and cognitive function [22].

The literature contains several studies on multicomponent programs with dual tasks, but there is limited evidence on their application in groups of older adults with low schooling levels, especially in Brazil. According to the Brazilian Institute of Geography and Statistics (IBGE), 60% of older people are illiterate [23], and these results are reinforced by the Functional Illiteracy Indicator (INAF) of Brazil, which reports that 53% of functional illiterates are aged between 50 and 64 years [24]. One study [25] obtained similar data in Northeastern Brazil, where 51% of older people had not completed elementary school. Moreover, most Brazilian studies on these programs were conducted in Southern and Southeastern Brazil involving community-dwelling older adults with higher schooling levels [26–28]. In this respect, promoting easy-to-understand assessments and exercise proposals that guarantee the adherence of older people with low schooling levels is a challenge in Northeastern Brazil.

Thus, given the limited number of studies that analyze the effects of multicomponent exercise programs in groups of community-dwelling older adults with low schooling levels in Northeastern Brazil, the aim of the present study was to analyze the effects of a dual-task multicomponent program compared to simple exercise training on the physical and hemodynamic functions of groups of community-dwelling older people from Northeastern Brazil with low schooling levels.

## 2. Materials and Methods

This is a pilot randomized clinical trial conducted according to CONSORT (Consolidated Standards of Reporting Trials) [29]. It was registered in the Brazilian Registry of Clinical Trials (ReBEC) under RBR-5gjrzz and follows the TIDieR (Template for Intervention Description and Replication) checklist [30]. The study was approved by the Research Ethics Committee of the Trairi School of Health Sciences (FACISA/UFRN) under protocol number 3.204.561.

### 2.1. Sample

Nonprobability convenience sampling was applied, and participants were recruited from the waiting list of the university's integrated school clinic and by disseminating the study on social media sites and a local radio program. Subjects were recruited between September and December 2019, contacted by telephone to explain the research, and invited to participate voluntarily. All participants provided written informed consent.

**Figure 1:**
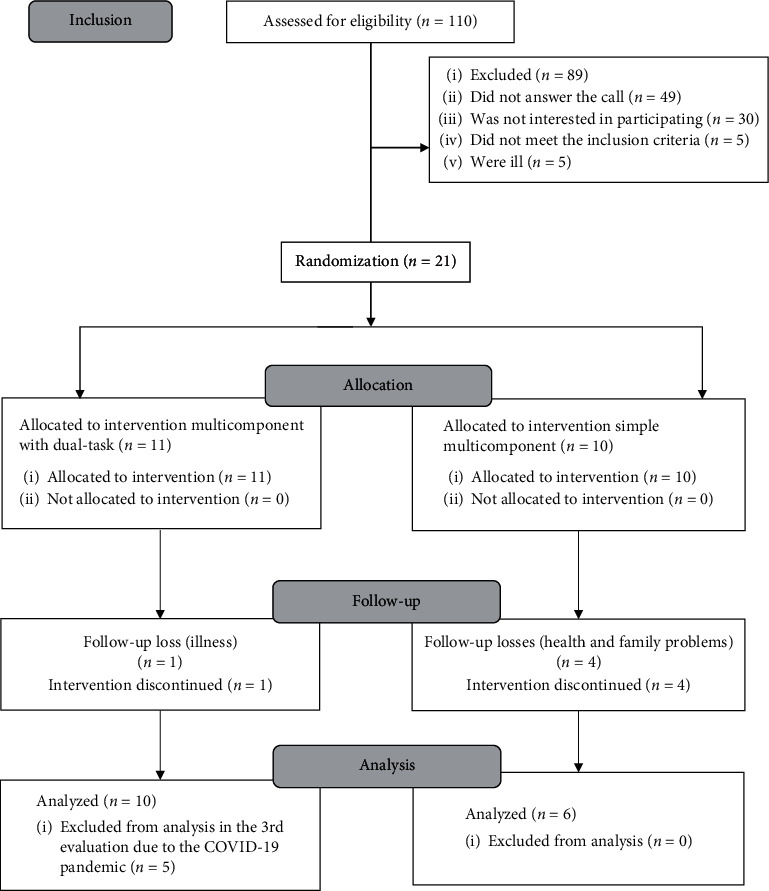
Study flowchart.

The following inclusion criteria were established: aged 60 years or older and of both sexes; moreover, participants were included according to schooling: illiterate, incomplete elementary education (up to grade 4), complete elementary education (up to grade 9), and complete secondary education (up to the 3rd year). Excluded were older adults with severe orthopedic disorders (upper limb fractures and amputation) and severe neurological diseases or those who had undergone physiotherapy in the previous two months. Individuals with less than 80% adherence to the interventions and scores greater than or equal to 22 points on the Leganés Cognitive Test (LCT) were also excluded.  Figure 1 presents the study flowchart.

### 2.2. Randomization, Allocation, and Blinding

Randomization was conducted by a blind researcher using http://www.randomization.com. After randomization and initial assessment, each individual responsible for the intervention received a folder with the randomized numbers allocated to one of the two groups: multicomponent with a dual task and the simple exercise group.

The subjects were functionally assessed individually by blinded examiners. The researcher in charge of assessments was blinded to group allocation, as were the participants themselves. The data collected during assessments were not divulged to the researchers and the participants were instructed not to share their experiences or information related to the intervention. Finally, the researcher in charge of statistical analysis conducted blind analysis.

### 2.3. Measurements

Assessments were conducted at three different times: first (pre), second (after the 3rd week), and third evaluations (follow-up). Functional data collection occurred on two days for sixty minutes. Initially, we applied a semistructured questionnaire to collect sociodemographic variables, followed by clinical-functional assessment, seven questionnaires, and functional testing. At the second meeting, force platform data were collected for 30 minutes. Test reliability was not determined.

#### 2.3.1. Hemodynamic Function

Systolic and diastolic blood pressure (SBP and DBP), heart rate (HR), and peripheral oxygen saturation (SpO2) were recorded before and immediately after each session on a follow-up chart using sphygmomanometer, stethoscope, and pulse oximeter measurements, respectively. Adverse effects and observations regarding the intervention were also recorded on the same chart.

#### 2.3.2. Functional Capacity

The Short Physical Performance Battery (SPPB) was validated and adapted for the Brazilian population [31]. The instrument is effective in assessing the lower limb performance of older adults. It is used to assess functional capacity by applying the following three tests: standing static balance; usual gait speed measured twice going back and forth on a 3 or 4-meter course; and lower limb strength with the subject sitting and rising from a chair five times without using their upper limbs. The final SPPB score is the sum of the three tests, which can range from 0 to 12 points, and individuals were classified as follows: 0 to 3 points, very poor performance; 4 to 6 points, low performance; 7 to 9 points, moderate performance; and 10 to 12 points, good performance. A cutoff point of 9 points is applied to identify frailty [32].

#### 2.3.3. Mobility, Falls, and Fear of Falling

The Timed Up and Go (TUG) test, created by Podsiadlo and Richardson [33], was validated for Brazilian older adults and aims to quantify mobility and assess the risk of falls in this population. The predictive value for falls in older adults is more than 12.4 seconds [34]. In order to undergo the test, the volunteers were instructed to rise from a chair, walk 3 meters, return to the chair, and sit down again.

In the present study, the TUG was applied three consecutive times with two variations, the first in a single modality. In the second and third applications, the TUG was performed by introducing a dual task. In the second test, we added a motor-motor task, consisting of subjects walking while carrying a disposable plastic cup 1/3 full of water and instructed not to spill the liquid; when spillage occurred, the test was repeated. Finally, the test with the motor-cognitive task was performed, where the individual walked while saying the names of animals. The time to complete each task was measured with a stopwatch.

The Falls Efficacy Scale-International-Brazil (FES-I-Brazil), an instrument adapted and validated in Brazil, contains questions on the fear of falling during 16 activities, with scores between one and four. The total score varies between 16 and 64; the smallest value corresponds to no fear of falling and the highest to extreme fear of falling during social activities and activities of daily living (basic and instrumental) and tasks related to postural control. A score ≥23 points suggests a history of sporadic falls, and a score ≥31 points indicates recurrent falls [35]. In addition, participants self-reported the number of falls in the last 6 months.

#### 2.3.4. Dynamic and Static Balance

The Figure of 8 Walk (F8W) test assesses dynamic balance, with individuals asked to walk a 10 m long and 15 cm wide figure of eight courses as fast as possible [36]. The test was performed three times, the first with no change and the second and third with a secondary task. The second test was conducted with the individual walking the course with a disposable plastic cup 1/3 full of water and asked not to spill any liquid. If spillage occurred, the test was repeated and in the last test, the subject walked the figure of eight courses while simultaneously saying the names of fruits.

Computerized posturography was conducted using a force platform (EMG System*®*) to measure body oscillation, body oscillation speed, and the area of body displacement measured by the center of pressure (COP) in relation to the support base [37]. To assess the effect of adding cognitive tasks on balance, participants remained barefoot on the force platform, staring at a target 1 meter away, with arms at their sides and feet 10 cm apart. The frequency was set at 100 Hz and the following measures were collected: average position in the anteroposterior (M-AP) and mediolateral (M-ML) axes and displacement from the COP area, during five standing conditions: no cognitive task, saying the names of animals or cities, words with a preestablished letter, performing calculations, and recalling figures.

In order to avoid the learning effect, some aspects of the activities were changed in the postintervention assessment, but the domains evaluated were the same, namely attention, verbal fluency, recall, and memory (Table 1). The order of activities was randomized using the Random Number® application (Android), a random number generator. Each task was performed in 60 seconds and the time to organize the program for the next task allowed the participant to rest, resulting in total task duration of thirty minutes. In the case of upper arm movement during posturography, imbalance, or imminent danger of falling on the force platform, the test would be stopped, the individual offered assistance and allowed to rest, and the test restarted.

#### 2.3.5. Cognitive Function

The LCT, created by Zunzunegui et al. 2000 [38] and validated in Brazil [39], was applied to conduct a quick and easy assessment of cognition, without the influence of schooling, thereby improving the screening of this population. The total score is 32 points, with the highest scores indicating better cognitive performance and the cutoff point for cognitive impairment being 22 points.

#### 2.3.6. Dual-Task Difficulties

The frequency with which individuals experience dual-task difficulties in their daily routine was assessed using the 10-item Dual-Tasking Questionnaire. This instrument contains 10 items classified on a 0–4 scale, where the answers vary between very often to never, or not applicable. For this instrument, the higher the score is, the more difficult it is to perform simultaneous tasks [40].

### 2.4. Interventions

The individuals underwent their respective interventions, using the adapted Ageing*-*ON DUAL-TASK program [41], which contains three multicomponent exercise phases, involving strengthening, balance, and dual tasks (Table 2).

The exercise program occurred between January and March 2020 and was adapted according to the participants' cultural and regional context, with further adjustments after a single-session pilot test. The following changes were made: program duration was shortened to 6 weeks, using strengthening and balance exercises and secondary tasks from the second month of the original intervention, without increasing exercise complexity. Cognitive tasks were applied in line with the first and second complexity levels of the original program [41]. Dumbbells and ankle weights were used for muscle strengthening, given that they are easy-to-use and low-cost materials for groups of older adults.

Group G1 underwent 8 sessions of multicomponent exercises with a dual task and G2 executed 8 sessions of simple exercises, with no cognitive tasks. All the exercises were demonstrated to both groups. After this intervention, the groups were submitted to a second evaluation. Finally, the two groups performed only 8 sessions of simple exercises and underwent a third evaluation at the end of these sessions (Table 2).

First, the examiners demonstrated each exercise to the group. Participants then performed the exercise program simultaneously, with the help of verbal commands and balance support in the standing position, when needed. The adapted program lasted 60 minutes for group 1 and 40 minutes for group 2, with a 1-minute rest between exercises. However, when the two groups performed only exercises without secondary tasks, the duration was 40 minutes. Thus, three evaluations and sixteen intervention sessions were held during the data collection period.

### 2.5. Statistical Analysis

The Statistical Package for the Social Sciences (SPSS) and GraphPad Prism were used for statistical analysis. The Shapiro-Wilk normality test identified nonnormal data distribution, requiring the use of nonparametric tests for inferential analysis. The descriptive analysis involved measures of central tendency (median), dispersion (1st and 3rd quartiles), and absolute and relative frequencies. Comparative analysis of qualitative variables was carried out with the chi-squared test. The Mann-Whitney test was applied to compare the intergroup values or differences in the quantitative variables in the first, second, and third evaluations. The Wilcoxon test was used to compare the 1st and 2nd and 1st and 3rd evaluations and the hemodynamic variables before and after intervention. Significance was set at *p* value < 0.05.

## 3. Results

A total of sixteen individuals completed 80% of the group intervention. Five G1 participants did not undergo the third evaluation, since its conclusion was precluded by the need for social isolation caused by the COVID-19 (SARS-COVID-2) pandemic. The results demonstrate no significant intergroup differences in sociodemographic characteristics in the first evaluation (Table 3). With respect to physical and cognitive function, the latter, which was assessed by the LCT, exhibited no intergroup difference, but the G2 score rose after the third evaluation (*p*=0.031). Intergroup frailty, as assessed by the SPPB, also improved (*p*=0.008) after the third evaluation, as well as mobility tested by the simple (*p*=0.011), motor (*p*=0.018), and cognitive (*p*=0.010) TUG test. For dynamic balance, the intergroup results were significant after the third evaluation in the simple (*p*=0.018), motor (*p*=0.022), and cognitive (0.018) F8W test. Moreover, the cognitive F8W test in G2 improved between the second and third evaluations (*p*=0.034). With respect to the difficulty in performing a dual task in the activities of daily living and the fear of falling (FES-I-Brazil), no intergroup differences were observed after the interventions (Table 4).

Posturography showed differences in the evaluations of each group (Table 5). In group 1, differences in body oscillations were observed between the 1st and 2nd evaluations in the anteroposterior position under the following conditions: semantic verbal fluency, calculations, and memory/attention; the mediolateral position exhibited a statistical difference in the eyes open condition. In group 2, significant differences were found between the 1st and 3rd evaluations of oscillation in the mediolateral position for eyes open, memory, and attention, total displacement showed differences in all the conditions, and in terms of the COP area, no intergroup differences were observed. Significant intergroup differences were found between the medians (between the 1st and 2nd and 1st and 3rd evaluations), especially the last two.

**Figure 2:**
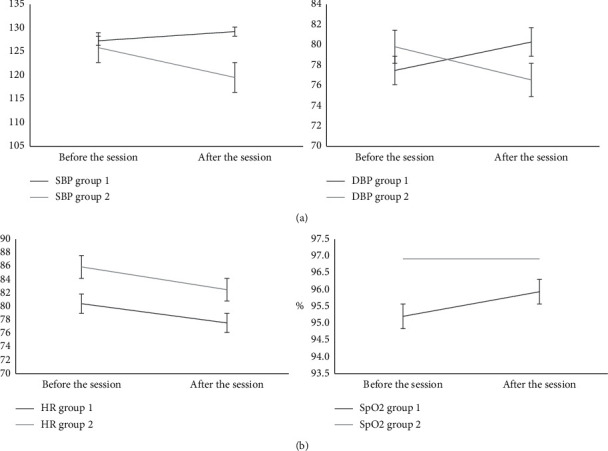
Variation between the medians of hemodynamic function before and after the intervention in both groups. SBP = systolic blood pressure; DBP = diastolic blood pressure; HR = heart rate; SpO_2_ = peripheral oxygen saturation; G1 = Group 1; G2 = Group 2.

The hemodynamic function of each group showed no significant median differences before and after each session for the following variables: SBP, DBP, HR, and SpO_2_ (Figure 2). The values of the variables were within the normal range for both groups.

## 4. Discussion

The aim of the present study was to compare the efficacy of an adapted dual-task multicomponent program and exercises without the addition of simultaneous tasks in groups of community-dwelling older adults with low schooling levels from Northeastern Brazil. According to the results, the dual-task multicomponent exercise program showed similar effects to those of a simple multicomponent exercise program. Several adjustments had to be made to the original multicomponent program due to the sociodemographic characteristics of the study population. These adjustments more effectively represented reproducibility, since they enabled better understanding by the participants. In addition, the group exercise approach was an important factor because it promoted social interaction and adherence to the program.

With respect to the dual tasks, the findings of this study demonstrated that adding this modality did not produce better results than simple exercises in community-dwelling older adults with low formal education. This indicates that the benefits will be similar, irrespective of whether the intervention adopted includes dual tasks or not. Another aspect to consider is the participants' schooling level, which may have influenced performance in exercises that demand greater attention. This is because low schooling level may have had an effect similar to the degree of difficulty among the interventions, independent of the amount of attention required by the exercises. Thus, schooling may be a confounding factor and may have influenced the absence of superior effects in the dual-task exercise program compared to single exercises.

The group that underwent simple multicomponent exercises (with no secondary task) showed positive behaviors after the intervention when compared to the group submitted to dual-task exercises. The results indicate an intergroup difference in mobility and dynamic balance between the first and third evaluations. Thus, the dual-task multicomponent exercise program demonstrated similar effects to those of its simple multicomponent counterpart in community-dwelling older adults. The results for all the 3rd evaluation variables in group 1 underwent changes due to sampling loss during the COVID-19 (SARS-COVID-2) pandemic, given that 4 individuals from this group were not assessed, despite having concluded the intervention.

Jehu et al. [20] analyzed community-dwelling older adults that underwent multicomponent training and found an improvement in mobility using the simple and dual-task TUG test. Similarly, a recent study by Purnamasari et al. [42] concluded that dual-task exercises reduced the risk of falls in older people. The dual-task multicomponent exercises executed by group 1 produced a positive result in dynamic balance, as determined by the F8W test in conjunction with a cognitive task. Simple multicomponent exercises demonstrated beneficial effects on the cognitive function of group 2, as observed in recent studies that applied multicomponent exercises in elderly adults [42–49].

The study groups were homogeneous in terms of sociodemographic profile and clinical and physiofunctional characteristics based on the measuring instrument data and mobility tests. Intergroup age and family income were also similar. Most of the sample of older adults were women, retired, sedentary, and with controlled systemic arterial pressure, similar to those of other studies with this type of intervention [12, 14, 21, 22, 50]. The results show that 75% of the older adults had low schooling levels (less than 4 years), and adaptations to the original multicomponent program were made, as described in the methods section. Similarly, a national study conducted by Ansai et al. [51] found that older adults had an average of 4.7 years of schooling.

In light of the low schooling levels in the present study, the LCT was selected for cognitive screening since it measures cognitive function and disregards the formal schooling of older persons. The cognitive assessment demonstrated no cognitive impairment in either group, according to the 22-point cutoff [39] given that this was an exclusion criterion. Studies that applied multicomponent training in older individuals obtained similar cognitive function findings to ours. In this respect, Gregory et al. [19] reported preserved cognitive function in older adults according to the Mini-Mental State Examination (MMSE). Other international studies [14, 15, 21] obtained similar adequate cognitive function results according to the MMSE of older adults aged 72.74 and 69 years, respectively.

Self-reports revealed that most of the older adults had memory problems and a low incidence of falls in the previous 6 months (none or 1). Although most did not report balance disorders, physical function, as assessed by the SPPB, showed reduced mobility and frailty for both groups. Collaborating the results of the present study, Câmara et al. [32] assessed 124 older subjects from Santa Cruz (Brazil) and Saint Bruno (Canada) and observed that those from Northeastern Brazil performed worse on the SPPB. In the same vein, Costa, Vieira, and Bento [45] found different prefrailty scores in community-dwelling older people.

Falls were screened in the present study, and the results obtained with FES-I-Brazil demonstrated that the older adults exhibited a history of sporadic falls and were concerned about this problem. Similar findings were reported by Borrás et al. and Wollensen et al. [12–21] who also found that the community-dwelling older individuals assessed by FES-1 in their studies were concerned about falling.

In the 1st evaluation, the TUG results of group 1 were higher than the cutoff point of 12.47 seconds for older adults (29) suggesting that this group was at greater risk of falling; on the other hand, the findings for group 2 were within the normal range. No significant intergroup difference in test duration was found in the 1st evaluation. The authors [9] and [15] assessed gait speed using TUG and found shorter times than that of the cutoff in older adults.

Gomes et al. [46] used dual-task exercises with sedentary older people, who obtained a score suggesting falls according to the FES I, and the TUG test with secondary tasks was executed faster than the simple test. When the TUG test with a secondary task (motor or cognitive) was compared, the groups exhibited similar behavior. In this respect, Fatori et al. [47] found no difference between simple and dual-task execution times (holding a plastic cup during the test) but did observe a difference between the simple test and TUG with bimanual activity (transferring coins), when the latter showed a significant increase in execution time.

Dynamic balance was assessed by the simple and dual-task F8W test (holding a glass of water while walking or saying the names of fruits). In the first assessment, both groups obtained times of more than 10.49 seconds, the average value reported by Hess et al. [48]. Both groups had difficulty walking in environments that required changing direction and speed. In addition, for the results assessed by the 10-item Dual-Tasking Questionnaire, the older adults of both groups experienced similar difficulty in performing two tasks simultaneously in their daily activities.

The hemodynamic function assessed by SBP and DBP, HR, and SpO_2_ showed no intergroup differences during the interventions, exhibiting normal values. The hemodynamic stability observed here suggests that the simple or dual-task multicomponent exercise program is safe for older people in Brazil. Gonçalves et al. [27] found that blood pressure declined in community-dwelling older adults in Brazil after multicomponent exercises, but HR showed no difference.

The study found a significant reduction in average anteroposterior position only in older individuals who were submitted to dual-task multicomponent exercises. There were no changes in the COP area in the standing position in posturography for either group. D. Jehu, Paquet, and Lajoie [20] observed that reaction time improved after the intervention in the group that performed balance, mobility and cognition exercises, but the COP and displacement did not change.

After the 2nd evaluation, both groups showed significant improvements in cognitive verbal fluency tasks performed during posturography assessments; however, phonetic verbal fluency exhibited no difference in any of the interventions. Group 1 performed the calculation task better after the 2nd evaluation and group 2 showed improved memory after the 3rd.

The difference of the present study is the investigation of group physical exercises in older Brazilians with low schooling levels. The subjects of both interventions accepted and adapted satisfactorily to the multicomponent program. Group exercises provided greater motivation, interaction, stimulation, and socialization between participants, resulting in better adherence to the interventions. In Brazil, older people enjoy being part of groups, especially family and religious gatherings and meetings with friends.

The following adaptations were made to enable an intervention involving older people with low schooling levels in Northeastern Brazil: changes to the exercise program (number of stages, materials, and group modality) and simple accessible cognitive tasks in posturographic assessment. The use of low-cost, easy-to-use materials facilitated applying the program in a group setting. One drawback of group exercises was the noisy environment, which can reduce the concentration and attention of the individuals.

The study limitation was the suspension of the 3rd evaluation due to the COVID-19 (SARS-COVID-2) pandemic, resulting in the exclusion of 5 group 1 individuals from this measurement stage. The older adults who participated in the research were from the community and the assessment and intervention environment was institutional (Physiotherapy School Clinic of the University). The groups did not meet in the clinic because the intervention times were different. The sample number was small, given that it was obtained from a limited list of Physiotherapy School Clinic patients from a public university. As a result, it was not possible to determine the optimal sample and effect size, nor was intention to treat measured, making this a pilot study.

## 5. Conclusions

The adapted multicomponent exercise program produced benefits for the community-dwelling older adults with low schooling levels, with no difference between the simple exercises and the dual-task modality. Hemodynamic stability, comprehension and adherence to the measurements and interventions, improved mobility, frailty, static postural control, and dynamic balance were exhibited by all study participants. The favorable results found via SPPB and TUG tests may be associated with greater independence, lower number of falls, and consequently, fewer fractures, hospitalizations, and disabilities, thereby contributing to better quality of life in community-dwelling older adults.

The possible influence of participants' low formal schooling and low income on the proposed interventions was taken into account. The changes in the program, namely, group exercises, the use of low-cost, easy-to-use materials, and simple cognitive tasks in posturographic assessment made the study possible and seem to have promoted the understanding, interest, and adherence of the older Brazilians with low schooling levels.

The results for all the variables in the 3rd assessment of group 1 were certainly affected by the sampling loss caused by the COVID-19 (SARS-COVID-2) pandemic, given that 4 individuals were not assessed, despite having concluded the intervention.

## Figures and Tables

**Table 1:** Description of associated tasks to assess dual-task performance on the force platform.

Cognitive domains	Posturography (associated tasks)	
	1st assessment	2nd and 3rd assessments
None	Standing with eyes open, and no cognitive task	Standing with eyes open, with no cognitive task
Semantic verbal fluency	Saying the names of animals (good performance >11 correct answers)	Saying the names of cities (good performance >11 correct answers)
Phonetic verbal fluency	Saying words that start with the letter “F”	Saying words that start with the letter “V”
Calculation	Calculations (100–1 for illiterates and 100–3 for the others)	Calculations (100–1 for illiterates and 100–3 for the others)
Memory and attention	Recalling (remembering 6 previously shown figures: elephant, padlock, flower, bird, banana, and sheep)	Recalling (remembering 6 previously shown figures: house, flower, rabbit, pineapple, cup, and fish)

**Table 2:** Description of the adapted program applied to each group [41].

G1-Multicomponent group with dual task	
5-minute warm-up	Upper and lower limb stretching
30 seconds for each limb	
2 exercises	
Upper limb exercises + cognitive task	Elbow flexion + naming the months of the year in order
1 set of 12 consecutive repetitions for each upper limb with a 1 kg dumbbell	Elbow extension + when the color presented is green, the subject says yellow, and vice versa
Lower limb exercises + cognitive task	Knee flexion + naming colors/days of the week/names
1 set of 12 consecutive repetitions for each lower limb with a 1 kg ankle weight	Knee extension + when a green card is shown, individuals raise their hand
Balance training 2 sets, 10 seconds for each exercise	On tiptoes +
	Naming the months of the year starting with a random month (if the individual says January, the second says February and so on until the time is up)
	Single-leg stance +
	The instructor says Yes and the individual says No, and vice versa
	Semitandem +
	The instructor says a word (house, ball, tree, and flower) while the individual holds a glass and a ball without letting them fall, repeating the word
	Reach for a ball that the instructor alternates in different directions
	Counting 10 + 1, 11 + 1, 12 + 1 + ….

G2: simple multicomponent group	
5-minute warm-up	Upper and lower limb stretching
30 seconds for each limb	
2 exercises	
Upper limb exercises	Elbow flexion
1 set of 12 consecutive repetitions for each upper limb with a 1 kg dumbbell	Elbow extension
Lower limb exercises	Knee flexion
1 set of 12 consecutive repetitions for each lower limb with a 1 kg ankle weight	Knee extension
Balance training 2 sets, 10 seconds	Tiptoe
	Single-leg stance
	Semitandem
	Reach for a ball that the instructor alternates in different directions

**Table 3:** Descriptive analysis of the sociodemographic profile of each group.

Variables	Group 1	Group 2	*p*-value^*∗*^
	Multicomponent with dual task (*n* = 10) *n* (%) or median (1Q; 3Q)	Simple multicomponent (*n* = 6) *n* (%) or median (1Q; 3Q)	
Age (years)	72 (65/80)	71 (65/72)	0.548
Sex (F/M)	8/2 (80%/20%)	6 (100%)	0.242

*Schooling*			0.411
** **Incomplete elementary (up to grade 4)	8 (80%)	4 (66.7%)	
** **Complete elementary (up to grade 9)	1 (10%)	2 (33.3%)	
** **Complete secondary (up to the 3rd year)	1 (10%)	0	

*Profession*			0.211
** **Retired	10 (100%)	6 (100%)	

*Family outcome*			0.238
Up to 1 MW	4 (40%)	3 (50%)	
Between 1 and 2 MW	5 (50%)	3 (50%)	
Between 4 and 7 MW	1 (10%)	0	

HTN (Y/N)	9/1 (90%/10%)	4/2 (66.7%/33.3%)	0.247
Diabetes (Y/N)	2/8 (20%/80%)	2/4 (33.3%/66.7%)	0.551
Sedentary (Y/N)	7/3 (70%/30%)	4/2 (66.7%/33.3%)	0.889
Smoker (Y/N)	0/10 (0%/100%)	0/6 (0/100%)	0.383
Alcohol consumer (Y/N)	0/10 (0%/100%)	0/6 (0/100%)	0.146

*Self-reported memory deficit*			
** **No	0	2 (33.3%)	0.146
** **Sometimes	8 (80%)	3 (50%)	
** **Frequently	2 (20%)	1 (16.7%)	

*Self-reported balance problem/disorder*			
** **No	2 (20%)	3 (50%)	0.383
** **Sometimes	7 (70%)	3 (50%)	
** **Frequently	1 (10%)	0	

*Fall frequency (6 months)*			
** **0	6 (60%)	4 (66.7%)	0.703
** **1	3 (30%)	2 (33.3%)	
** **2	1 (10%)	0	

*Q* = quartile; *n* = number; *F* = female; *M* = male; MW = minimum monthly wage (≈USD200.00); *Y* = yes; *N* = no; HTN = hypertension ^*∗*^*p*-value obtained by the Mann-Whitney test to compare quantitative variables or the chi-squared test to compare qualitative variables.

**Table 4:** Measurement instruments and functional tests in the three assessments.

Variables	Group 1	Group 2	*p*-value^*∗∗*^
	Multicomponent with dual task (*n* = 10)	Simple multicomponent (*n* = 6)	
	Median (1Q; 3Q)	Median (1Q; 3Q)	
LCT 1st ev.	25 (23.7; 27.7)	25 (23.5; 28)	0.913
LCT 2nd ev.	27 (25.7; 30.2)	26 (24.2; 31.2)	0.622
LCT 3rd ev.	30 (28.5; 32)	29 (25.5; 31.2) *∗* (*p*=0.031)	0.459
DT-Q 1st ev.	19 (15.5; 23.2)	15 (12.7; 19.2)	0.157
DT-Q 2nd ev.	18 (14.7; 22.5)	15 (13.7; 19)	0.156
DT-Q 3rd ev.	18 (14.4; 20.5)	16 (11.7; 20)	0.521
FES-I-Brazil 1st ev.	28 (21.7; 35.2)	27 (21.7; 35.7)	0.913
FES-I-Brazil 2nd ev.	27 (24.5; 35.2)	26 (20.7; 38.5)	0.745
FES-I-Brazil 3rd ev.	30 (23.5; 44)	27.5 (22.5; 34.7)	0.647
SPPB 1st ev.	7 (5.7; 8.2)	8 (7; 8.5)	0.165
SPPB 2nd ev.	7.5 (6.7; 8)	8 (7; 9.2)	0.308
SPPB 3rd ev.	7 (7; 8)	10 (9.5; 10.2)	0.008^*∗∗*^
Simple TUG 1st ev.	13.1 (11.8; 15.8)	11.3 (10.5; 14.9)	0.329
Simple TUG 2nd ev.	13.4 (10.5; 15.2)	12.1 (10.3; 13.8)	0.416
Simple TUG 3rd ev.	15.4 (13.2; 20.5)	10.8 (10.4; 11.8)	0.011^*∗∗*^
Motor TUG 1st ev.	15.7 (12.5; 17.4)	12.4 (11.4; 15.4)	0.278
Motor TUG 2nd ev.	14.4 (10.7; 18.4)	12 (10.5; 14)	0.328
Motor TUG 3rd ev.	19.1 (13.9; 21.1)	11.2 (10.5; 13.4)	0.018^*∗∗*^
Cognitive TUG 1st ev.	15.3 (13.4; 17.9)	13.7 (11.2; 16.1)	0.254
Cognitive TUG 2nd ev.	15.3 (13.4; 17.9)	13.6 (11.4; 15.1)	0.356
Cognitive TUG 3rd ev.	21.8 (16.2 22.9)	13.6 (11.4; 15.1)	0.010^*∗∗*^
Simple F8W 1st ev.	15.5 (12.9; 17.6)	12.8 (11.5; 14.7)	0.480
Simple F8W 2nd ev.	15.5 (13.6; 19.3)	14.3 (10.8; 15.5)	0.212
Simple F8W 3rd ev.	19.7 (16.2; 24.2)	12.8 (11.5; 14.7)	0.018^*∗∗*^
Motor F8W 1st ev.	16.5 (14.4; 18.1)	13.5 (12.5; 15.6)	0.065
Motor F8W2nd ev.	16.6 (13.4; 19.7)	13.5 (12.5; 15.6)	0.129
Motor F8W 3rd ev.	21.5 (16.6; 25.4)	13.5 (12.5; 15.6)	0.022^*∗∗*^
Cognitive F8W 1st ev.	16.6 (14.7; 19.1)	13.6 (12.1; 15.7)	0.175
Cognitive F8W 2nd ev.	15.6 (14.6; 23.4)	14.9 (13.5; 16)	0.278
Cognitive F8W 3rd ev.	23.1 (17.4; 31.2) *∗* (*p* = 0.043)	13.6 (12.1; 15.7)	0.018^*∗∗*^

*n* = number; 1st *Q* = first quartile; 3rd *Q* = third quartile; 1st ev. = first evaluation; 2nd ev. = second evaluation; 3rd ev. = third evaluation; LCT = Leganés Cognitive Test; DT-Q = 10-item Dual-Tasking Questionnaire; FES-I-Brazil = Falls Efficacy Scale-International-Brazil; SPPB = Short *Physical Performance Battery*; TUG = Time Up and Go Test; F8W = Figure of 8 Walk Test; motor: test performed with the addition of a motor task; cognitive: test performed with the addition of a cognitive task; ^*∗*^*p*-value to compare 1st and 3rd evaluations by the Wilcoxon test; ^*∗∗*^-value for intergroup comparison by the Mann-Whitney test.

**Table 5:** Posturographic variations between the first, second, and third evaluations.

Variables	Group 1			Median differences *p* value^*∗∗∗*^		Group 2			Median differences *p* value^*∗∗∗*^	
	Multicomponent with dual task (n = 10)					Simple multicomponent (n = 6)				
	Median (1Q; 3Q)					Median (1Q; 3Q)				
	1st ev.	2nd ev.	3rd ev.	Diff.	Diff.	1st ev.	2nd ev.	3rd ev.	Diff	Diff
				1st-2nd ev.	1st-3rd ev.				1st-2nd ev.	1st-3rd ev.
*AP position (cm)*										
Eyes open	47 (45.7; 61)	46 (6; 48.2)	59 (32.5; 59)	0.01	-0.24	54 (36.7; 61.2)	47 (46; 50.7)	32.5 (6; 59)	−0.13	−0.01
Semantic verbal fluency	47 (45.7; 61)	25 (6; 46.2)*∗*(*p*=0.035)	59 (4.5; 59)	0.01	-0.24	53.5 (36; 61.2)	47 (33; 47.2)	6 (6; 59)	−0.13	−0.005
Phonetic verbal fluency	47 (45.7; 61)	47 (6; 48)	59 (32.5; 59)	0.015	−0.24	53.5 (35.2; 61.2)	44.5 (34.2; 51.5)	6 (6; 59)	−0.11	−0.005
Calculations	47 (46.7; 61)	45.5 (6; 50)*∗*(*p*=0.043)	59 (32.5; 59)	−0.005	−0.24	54.5 (36; 61.2)	46 (44; 50.7)	6 (6; 59)	−0.14	−0.005
Memory and attention	53 (46; 61)	44.5 (6; 47.5)*∗*(*p*=0.011)	59 (32.5; 59)	−0.15	−0.24	61 (46.7; 61.2)	46.5 (35.7; 51.5)	6 (6; 59)	−0.12	−0.01

*ML position (cm)*										
Eyes open	24.5 (5; 49)	47 (43; 48.7)*∗*(*p*=0.039)	45 (45; 46.5)	−0.03	−0.27	48 (47.7; 49.5)	50 (46.7; 52.2)	46 (45.7; 47)^*∗∗*^(*p*=0.031)	0.015	−0.02^*∗∗∗*^ (*p*=0.033)
Semantic verbal fluency	49 (34.2; 49.5)	45 (42.5; 49.7)	45 (45; 46)	−0.045	−0.28	48.5 (36.5; 49.5)	46.5 (5; 51)	46 (45.7; 47)	0.015	−0.02^*∗∗∗*^ (*p*=0.043)
Phonetic verbal fluency	49 (34.2; 51)	46 (43.2; 51.5	46 (45; 46)	−0.02	−0.28	48 (36.5; 49)	48 (35.7; 51.5)	46 (45.7 47)	0.01	−0.02^*∗∗∗*^ (*p*=0.019)
Calculations	49 (34.2; 51)	44.5 (32; 49.5)	45 (45; 46)	−0.035	−0.28	48.5 (36.5; 49)	51.5 (35.7; 53.7)	46 (45.7; 47)	0.03^*∗∗∗*^ (*p*=0.049)	−0.02^*∗∗∗*^ (*p*=0.022)
Memory and attention	48.5 (5; 49.5)	44 (5; 47.2)	46 (45; 46.5)	−0.025	−0.27	48.5 (47.7; 51)	25.5 (5; 48.7)	46 (45.7; 47) ^*∗∗*^(*p*=0.031)	0.01	−0.02

*TD (cm)*										
Eyes open	30190 (5679; 6507)	52551 (5357; 111957)	2586 (1930; 3953)	238.37	522.51	6094 (5430; 6685)	124250 (92920; 158388)	4287 (3345; 4994)^*∗∗*^(*p*=0.031)	1.167.97	−19.1
Semantic verbal fluency	52575 (5120; 65786)	10575 (5483; 110120)	2697 (1950; 4082)	237.37	513.56	6228 (3530; 62108)	128796 (87972; 182224)	4044 (1593; 4506) “^*∗∗*^(*p*=0.031)	1.160.39	−17.93
Phonetic verbal fluency	29762 (5488; 62852)	55152 (5438; 115407)	2839 (1992; 3994)	248.31	−514.41	6154 (5255; 65182)	124015 (888910; 180530)	4298 (1597; 5104) ^*∗∗*^(*p*=0.031)	1.187.52	−13.68
Calculations	28881 (6102; 58851)	54376 (4572; 110106)	2885 (2134; 4162)	182.09	−495.93	6190 (5314; 63396)	125243 (83821; 172651)	3994 (1680; 4724) ^*∗∗*^(*p*=0.031)	1.151.8	−15.46
Memory and attention	6125 (2021; 58408)	11466 (5537; 111221)	3091 (2120; 4300)	313.78	−284.35	6328 (5040; 64392)	128114 (86888; 175620)	3121 (444.0; 5196) ^*∗∗*^(*p*=0.031)	1.173.83	−11.13

*Area (cm* ^*2*^)										
Eyes open	51 (1.00; 94.5)	139 (0.75; 368)	0 (0; 0.5)	1.24	−0.85	1 (1.0; 43.0)	461 (333.8; 741.3)	0.50 (0; 1.0)	4.6	−0.01
Semantic verbal fluency	85.5 (1.00; 126.8)	153 (0.750; 368)	0 (0; 0.5)	1.31	−0.86	1 (1; 113.8)	482 (303.3; 963)	1 (0; 1.0)	4.81	−0.005
Phonetic verbal fluency	45.5 (1.00; 118)	5.00 (0.75; 383)	0 (0; 0.5)	1.24	−0.83	1 (1; 51.7)	443 (303.8; 962.5)	1 (0; 1)	4.4	−0.005
Calculations	13.0 (1.00; 89.7)	54.5 (1.00; 298)	0 (0; 0.5)	0.9	−0.79	1 (1; 40)	458 (272.3; 873.8)	1 (0; 1)	4.57	−0.005
Memory and attention	41 (1.00; 103.5)	21 (0.75; 363)	0 (0; 0.5)	1.44	−0.41	1 (1; 122)	465 (288.8; 901.3)	1 (0; 1)	4.64	−0.005

*n* = number; 1st *Q* = first quartile; 3rd *Q* = third quartile; 1st ev. = first evaluation; 2nd ev. = second evaluation; 3rd ev. = third evaluation; Diff = difference between the medians; AP = anteroposterior; ML = mediolateral; TD = total displacement; cm = centimeter; cm2 = centimeter squared; ^*∗p*^-value to compare between the 1st and 2nd evaluations by the Wilcoxon test; ^*∗∗*^-value to compare between the 1st and 3rd evaluations by the Wilcoxon test; ^*∗∗∗*^*p*-value obtained by the Mann-Whitney test to compare the intergroup differences between the 1st and 2nd and 1st and 3rd evaluations.

## Data Availability

The data used to support the findings of this study are included in the article.
